# Integration of phytotherapy and chemotherapy: Recent advances in anticancer molecular pathways

**DOI:** 10.22038/IJBMS.2023.69979.15222

**Published:** 2023

**Authors:** Rouhollah Hemmati Bushehri, Parnian Navabi, Amir Mohammad Saeedifar, Nafiseh Keshavarzian, Negin Hosseini Rouzbahani, Ghasem Mosayebi, Ali Ghazavi, Khodayar Ghorban, Ali Ganji

**Affiliations:** 1 Department of Immunology, School of Medicine, Arak University of Medical Sciences, Arak, Iran; 2 Department of Immunology, Medical School, Aja University of Medical Sciences, Tehran, Iran; 3 Molecular and Medicine Research Center, Arak University of Medical Sciences, Arak, Iran; 4 Traditional and Complementary Medicine Research Center (TCMRC), Arak University of Medical Sciences, Arak, Iran

**Keywords:** Cancer, Chemotherapy, Combination therapy, Herbal medicine, Molecular pathways

## Abstract

Cancer is a disease characterized by abnormal and uncontrolled growth of cells, leading to invasion and metastasis to other tissues. Chemotherapy drugs are some of the primary treatments for cancer, which could detrimentally affect the cancer cells by various molecular mechanisms like apoptosis and cell cycle arrest. These treatment lines have always aligned with side effects and drug resistance. Due to their anticancer effects, medicinal herbs and their active derivative compounds are being profoundly used as complementary treatments for cancer. Many studies have shown that herbal ingredients exert antitumor activities and immune-modulation effects and have fewer side effects. On the other hand, combining phytotherapy and chemotherapy, with their synergistic effects, has gained much attention across the medical community. This review article discussed the therapeutic effects of essential herbal active ingredients combined with chemotherapeutic drugs in cancer therapy. To write this article, PubMed and Scopus database were searched with the keywords “Cancer,” “Combination,” “Herbal,” “Traditional,” and “Natural.” After applying inclusion/exclusion criteria, 110 articles were considered. The study shows the anticancer effects of the active herbal ingredients by inducing apoptosis and cell cycle arrest in cancer cells, especially with a chemotherapeutic agent. This study also indicates that herbal compounds can reduce side effects and dosage, potentiate anticancer responses, and sensitize cancer cells to chemotherapy drugs.

## Introduction

A cancerous condition occurs when cells grow uncontrollably and affect adjacent tissues. It is the second leading cause of death worldwide, seriously threatening the public health of all communities (1). According to a report by the World Health Organization, there were 19.3 million new cancer cases in 2020 and almost 10.0 million cancer deaths (2). There are many treatments for cancer worldwide, including surgery in the early stages of a tumor, radiotherapy, immunotherapy, chemotherapy, etc., each with its limitations (3). Due to the severe complications caused by the above-mentioned conventional therapies, scientists are trying to use herbal treatments besides conventional cancer therapies. People generally believe that herbal remedies have fewer side effects, are safer, and possibly less addictive, so they are the most commonly used group of therapies (4). 

**Figure 1.**
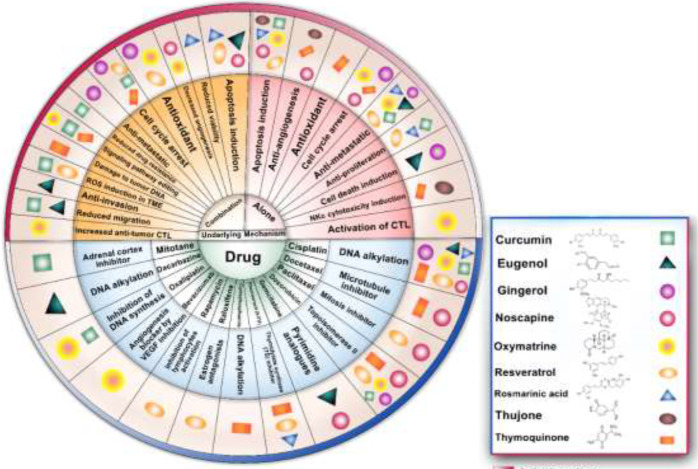
Antitumor effects of herbal compounds and combination with chemotherapy in cancer

Since ancient times, herbs and derivatives have been effective and safe treatments for many inflammatory diseases. Numerous clinical publications reported the beneficial effects of herbal medicines on survival, immune system regulation, and increasing cancer patients’ quality of life in cancer treatment (5). Hence, the anticancer activity of herbal medicines is attributed to the increase in autophagy cell death, apoptosis, tumor microenvironment modification, and suppression of tumor angiogenesis. It has been considered for decades to use herbal and anticancer drugs to overcome their limitations and minimize their side effects. It is also expected that such a combination therapy offers great potential to show synergistic effects in treatment (6). In this review article, we will discuss the impact of the active ingredients of several medicinal plants (Table 1), alone or in combination with chemotherapy drugs (Table 2), on cancer cells and their underlying mechanisms (Figure 1).

## Methods

**Figure 2:**
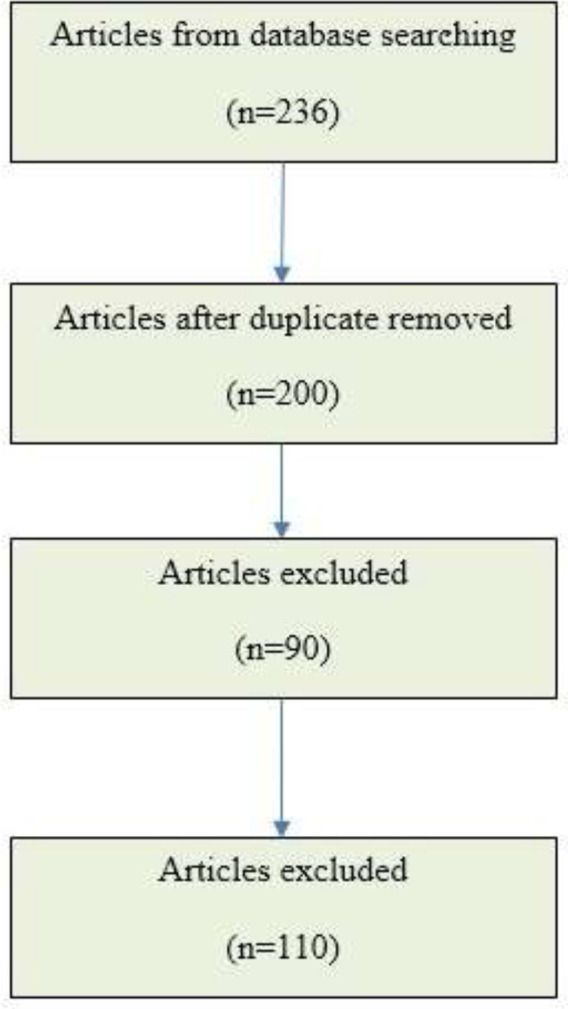
Study flowchart based on PRISMA. In this study from 2000 to 2022, 110 articles were chosen from 236 reviewed articles according to inclusion/exclusion criteria

The articles were searched based on the title and abstract using PubMed and Scopus databases with the keywords “Cancer,” “Combination,” “Herbal,” “Traditional,” and “Natural” from 2000 to 2022, which found 236 articles. Finally, 110 articles were chosen according to inclusion/exclusion criteria. Inclusion criteria were anticancer effect, antitumor effect, immune stimulation, immune mechanisms, and chemotherapy agent. The exclusion included duplicate articles and herbal use for cosmetic and hygienic products (Figure 2).

***Noscapine***

**Figure 3:**
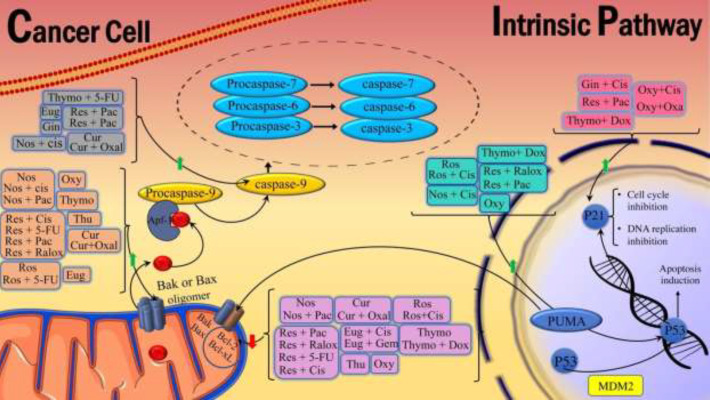
Natural herbal compounds and their combination with chemotherapy drugs affect the intrinsic signaling pathways of apoptosis

Noscapine is a benzylisoquinoline alkaloid isolated from Opium poppy with no hypnotic or euphoric effects. It has a low potential for addiction and is often used as an analgesic (Figure 2). It is currently a candidate for cancer treatment. A study on OVCAR4 ovarian cancer cells found that noscapine reduced the viability of cancer cells by down-regulating Bcl-xL and up-regulating caspase-3 (17). It also increases cell death in SW480 colon cancer cells by increasing the release of cytochrome C and reducing Bcl-xL and Bcl-2 gene expression (Figure 3) (18).

Noscapine derivatives inhibited angiogenesis by reducing the expression of HIF-1a and VEGF. In addition, noscapine suppresses the formation of new blood vessels and the proliferation of human umbilical vein endothelial cells (HUVEC-CS) (19). Noscapine suppresses the S phase in C6 glioblastoma cells by binding to β-tubulin and intermitting mitosis and apoptotic effect by phosphorylation of the anti-apoptotic protein Bcl-2 (Figures 3 and 5) (20).

***Noscapine and cisplatin***

**Figure 4:**
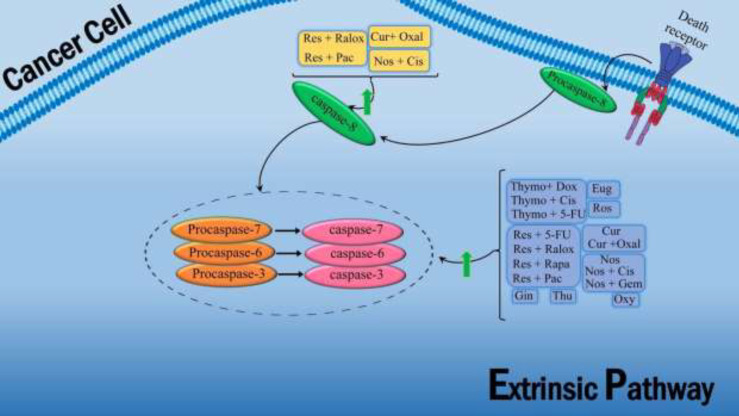
Natural herbal compounds and their combination with chemotherapy drugs affect the extrinsic apoptosis pathway

As an FDA-approved anticancer drug, cisplatin is more effective when used synergistically with noscapine, which allows it to be used at lower doses and minimize toxic side effects. There are some *in vitro* and *in vivo* experiments on human glioblastoma cells (U87MG) and lung cancer cells (A549 and H460) in response to the combination of noscapine and cisplatin (21, 22). The results showed that combination treatments on U87MG cells significantly decreased Ki67 molecules and increased apoptosis through caspase-3 and activated PARP levels (Figure 4). Furthermore, the combination of noscapine and cisplatin leads to markedly increased apoptosis in lung cancer cells compared to single treatments, as shown by increased expression of p53, p21, caspase-3, caspase-8, caspase-9, and PARP and decreased expression of Bcl2 (Figures 3 and 4). In a xenograft mouse model of lung cancer, treatment with this combination results in a more significant reduction in tumor volume than cisplatin and noscapine alone (23). In general, noscapine enhances the anticancer activity of cisplatin by activating several signaling pathways, like apoptosis.

The studies show a new function of noscapine in increasing the susceptibility of cisplatin-resistant ovarian cancer cells. Noscapine inhibits HIF-1α (a hypoxia-1-inducing agent), which is associated with drug resistance in solid tumors, cell proliferation, anti-apoptotic XIAP, and NF-κB. It increases the expression of caspase-3, which leads to increased sensitivity of cisplatin-resistant ovarian cancer cells (23, 24). *In vivo* experiments also showed that the combination therapy of noscapine and cisplatin increased the rate of tumor cell apoptosis in the xenograft mouse model and prevented tumor growth (24). These studies offer a new strategy to improve the effectiveness of chemotherapy on cisplatin-resistant human ovarian cancer.

***Noscapine and docetaxel***

A combination of noscapine and docetaxel (DTX) was studied in different animal models and breast cancer cell lines to increase efficacy, reduce the dose of the chemical agent, induce apoptosis, and reduce toxicity. Accordingly, it was shown that the combination of noscapine and DTX in a mouse model of triple-negative breast cancer (TNBC) was associated with increased levels of p38 and JNK, a reduction in anti-apoptotic proteins, as well as increased levels of DTX cytotoxicity. Low-dose exposure to noscapine sensitizes TNBC cells to DTX, increasing DTX cytotoxicity. 

So, using noscapine in conjunction with DTX can maximize the toxicity of DTX in the tumor and reduce the dose of DTX and dose-dependent adverse effects (25). Various studies have examined the combined use of noscapine and DTX in breast cancer and concluded that this combination offers more significant therapeutic benefits than single treatments. Therapeutic effects are achieved by interfering with cell cycle progression, causing apoptosis and reducing tumor size (25, 26).

***Noscapine and paclitaxel***

The combination of paclitaxel and noscapine increases apoptosis induction in cancer cells. In this respect, Robesia *et al*. showed that combining noscapine and paclitaxel in a human prostate cancer cell line (LNCaP and PC-3) significantly increased apoptosis compared to a single treatment by decreasing Bcl-2 and increasing Bax (Figure 3). It also inhibited the expression of androgen receptor (AR) and prostate-specific antigen (PSA) compared to a single treatment in these cells. Therefore, the combination of noscapine and paclitaxel can synergistically improve the effectiveness of prostate cancer treatment. Additionally, noscapine effectively inhibits proliferation and induces apoptosis in paclitaxel-sensible and paclitaxel-resistant human ovarian cancer cells via the JNK pathway (27, 28).

***Noscapine and doxorubicin***

Inactivating NF-KB and anti-angiogenic pathways is the mechanism by which noscapine enhances the anticancer activity of doxorubicin against cancerous tumors. Various studies have evaluated the combination therapy of noscapine and doxorubicin for tumor treatment. A study on a mouse model and a cell line of TNBC breast cancer revealed that the combined use of this compound reduced tumor volumes, decreased NF- κB VEGF expression, and increased apoptotic proteins compared with a single dose of this compound (29). 

The antitumor effects of noscapine and doxorubicin on 4T1 cells and the mouse model of breast cancer and the use of this compound in a nanocarrier coated with CREKA peptide in another study showed that this combination effectively reduced tumor growth in the mouse model. Also, the use of this compound on breast cancer cells showed more cytotoxicity than the use of these two substances alone (30). Based on the results of these studies, it is concluded that combination therapy with noscapine and doxorubicin, through NF-κB inactivation, apoptosis, and inhibition of angiogenesis, is more effective in treating tumors with minimal side effects when compared to chemotherapy alone.

***Thymoquinone***

Thymoquinone is a phytochemical compound found in the *Nigella sativa* with anti-inflammatory and anti-oxidant effects (Figure 2). Animal and cell studies have shown that thymoquinone has anti-inflammatory, antimicrobial, antiparasitic, anti-oxidant, antihyperglycemic, and anticancer properties. It was found that thymoquinone decreased cell viability and promoted apoptosis in A546 human lung tumor cells. Additionally, the Bax/Bcl-2 ratio has increased significantly in the lung cancer cells treated with thymoquinone (Figure 3) (31). 

Thymoquinone has been shown to induce reactive oxygen species (ROS) production, GSH reduction, and mitochondrial dysfunction which induce apoptosis and decrease proliferation in C6 glioma cells (32). Besides, thymoquinone suppressed hepatocellular carcinoma (HCC) in the mice model by induction of TRAIL-mediated apoptosis, suppressing TGF-β1 expression, and reducing oxidative stress (33). Scientists suggested that thymoquinone enhanced natural killer cell cytotoxicity and activities after an experiment on leukemia mice. Anti-leukemic effects of thymoquinone were examined in the WEHI-3 murine leukemia cell line, in which thymoquinone increased early apoptosis in the WEHI-3 cells. It could inhibit WEHI-3 growth in the mice model and highlighted the potential of thymoquinone to be developed as an anti-leukemic agent (33).

***Thymoquinone and 5- fluorouracil (5-FU)***

Cancer stem cells (CSCs) resident in cancerous tissues of the colon contribute to chemotherapy resistance and disease recurrence. It is well known that the survival of colonic CSCs after treatment with 5-FU leads to cancer recurrence. The study on CSCs showed that the combination of 5-FU and thymoquinone reduced the signaling pathways of WNT/ß-Catenin and PI3K/AKT in HCT116 colorectal cancer cells, and transcriptional activity of ß-Catenin and cell adhesion effectively reduced the angiogenic capacity of the remaining resistant tumor cells (34). 

Other studies have demonstrated these antitumor activities by suppressing Wnt, β-catenin, and NF-κB expression in a mouse model of colorectal cancer (35). The combined treatment of 5-FU and thymoquinone in a mouse model of gastric cancer demonstrated increased activation of caspase-3 and caspase-9, leading to a significant increase in apoptosis in gastric cancer cells compared to thymoquinone alone (Figure 4) (36).

***Thymoquinone and cyclophosphamide***

**Figure 5:**
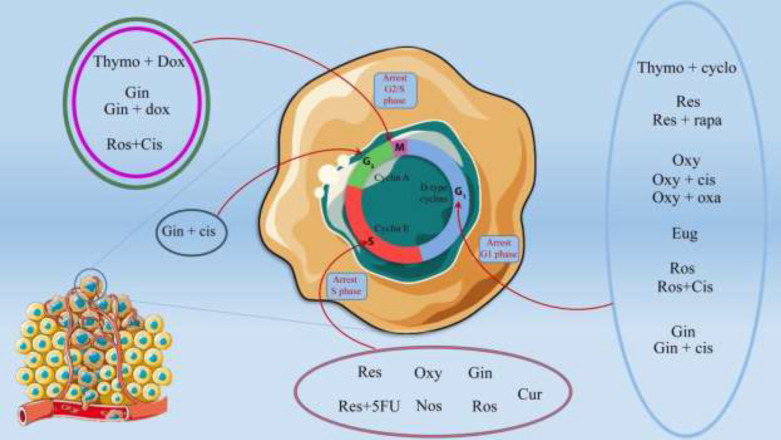
Overall view of the effects of herbal compounds and their combination with chemotherapy drugs on cell cycle arrest in the tumor cells

Cancer treatment with cyclophosphamide is associated with significant toxic effects due to the overproduction of reactive oxygen species (ROS) and increased levels of oxidative stress. Numerous studies have been done to minimize these harmful effects using thymoquinone. These studies suggest that thymoquinone can reduce toxicities induced by cyclophosphamide, such as hepatotoxicity, nephrotoxicity, toxicity on fertility, genotoxic damage in human lymphocytes, hemorrhagic cystitis, changes in cell differentiation and proliferation during fetal growth through induction of anti-oxidant mechanisms (37, 38). Besides its protective effects against toxicity, thymoquinone, combined with cyclophosphamide, improves the efficacy of anticancer drugs. A study on breast cancer cell lines found that combining thymoquinone and cyclophosphamide, which arrests the cell cycle in the G1 phase, reduces cyclin D1 expression, decreases PI3K/Akt signaling, increases the effectiveness of cyclophosphamide, and reduces its dosage in Her2-SKBR-3 and Her2-MDA-231 cells (Figure 5) (39). 

***Thymoquinone*** ***and cisplatin***

Various studies have investigated the synergistic effect of thymoquinone with cisplatin. Thymoquinone exerts several antitumor effects on cells by inhibiting NF-κB and increasing DNA damage. Based on the combination of thymoquinone and cisplatin studied in a mouse model and ovarian cancer cells (ID8-NGL), these compounds increased the effects of cisplatin by increasing DNA damage and cell apoptosis and reducing cell proliferation in ovarian cancer (40). 

Several studies have investigated the effect of cisplatin in combination with thymoquinone in cancer models. Based on an analysis performed on a xenograft mouse model of gastritis tumors, administering these combinations by increasing apoptotic factors and caspases resulted in significant tumor suppression compared to using agents alone. Thymoquinone also significantly increases the antitumor effects of cisplatin on gastric cancer by inhibiting the PI3K/AKT signaling pathway (41). In another study, the combined use of cisplatin with thymoquinone on hepatocellular carcinoma showed that thymoquinone modulates the GRP78/ CHOP/caspase 3 pathway increases the effect of cisplatin in the treatment of HCC and reduces its hepatotoxicity (Figure 4) (39). *In vitro* and *in vivo* studies on ovarian cancer showed that thymoquinone/cisplatin had the best inhibitory effect on cancer cell proliferation and increased apoptosis in tumors, thus reducing the overall tumor burden (40). 

Thymoquinone, cisplatin, and their combination were examined in a study against the human head and neck squamous cell carcinoma against normal oral epithelial cells. An equal portion of thymoquinone and cisplatin showed higher apoptosis in head and neck cancer cells (UMSCC-14C) than in healthy oral squamous cells and reduced cisplatin cytotoxicity on normal cells (40). These studies on cisplatin/thymoquinone combination have shown that activating apoptotic pathways, DNA targeting, and NF-ᴋb inhibition can enhance cisplatin apoptosis, reduce its cytotoxicity, and decrease cell proliferation that is resulting in tumor cell apoptosis. 

***Thymoquinone and doxorubicin***

An *in vitro* study of various tumors treated with a combination of thymoquinone and doxorubicin showed that thymoquinone increased the anticancer effects of doxorubicin by increasing apoptosis. Studies on hepatocellular carcinoma cells (HCC HepG2, SMMC-7721, and Huh7) have demonstrated that thymoquinone synergistically improves the anticancer activity of doxorubicin by activating caspases and reducing Bcl-2 expression (Figures 3 and 4). In contrast, they have relatively low toxicity to normal hepatocytes (HL-7702) (42, 43). 

Thymoquinone can increase the anticancer activity of doxorubicin and reduce its dose through ROS-dependent mechanisms. Combined use of thymoquinone and doxorubicin against adult T-cell leukemia cells (HTLV-1 positive (HuT-102) and HTLV-1 negative (Jurkat) CD4 + malignant T-cell lines), HL-60 human leukemia cells, and 518A2 melanoma cells, significantly inhibits the survival of these cells and increases their cell death by ROS-dependent or caspase-dependent mechanism compared to single-use of doxorubicin (44). The results of the apoptotic effect of thymoquinone on colorectal cancer (HCT116) and breast cancer cells (MCF7) treated with doxorubicin showed a higher sensitivity of these cells to apoptosis (45). 

Various studies have shown that using thymoquinone increases the effectiveness of doxorubicin even against resistant cells. Adding thymoquinone *in vitro* to doxorubicin-resistant MCF-7 breast cancer cells can inhibit these cells’ growth, proliferation, and apoptosis by increasing P53, P21 levels, and PTEN signaling factors, arresting the cell cycle in the G2/M phase and inhibiting Akt phosphorylation (Figures 3 and 5) (43). 

Animal studies show that thymoquinone enhances the therapeutic effect of doxorubicin by increasing its antitumor activity or reducing its toxicity to normal cells. Thymoquinone combined with doxorubicin can suppress tumor growth in the ATL xenograft mouse model, mainly by inhibiting tumor proliferation and increasing the apoptotic response, decreasing Ki67 expression, and increasing TUNEL positives cells in different sections of the tumor, more than in single therapies (46). 

Today, nanotechnology has shown many advantages in drug delivery in cancer treatment. According to various studies’ findings, using cockle shell-derived aragonite calcium carbonate nanoparticles, containing thymoquinone and doxorubicin, on breast cancer cells (MDA MB231) increased apoptosis significantly and decreased migration and cell invasion. Also, the prepared nanoparticles containing thymoquinone and doxorubicin can increase apoptosis, reduce ALDH activity, surface expression of CD44 and CD24, cell migration, and cell invasion on breast cancer stem cells compared to the drug-loaded single nanoparticles and drugs without biological transmission systems (47). Nano-formulation of doxorubicin and thymoquinone coated with Poly-N-acetylglucosamine (pGlcNAc) nanofibers on MCF-7 and HEPG2 liver carcinoma cells in mice significantly increased apoptosis, caspase-3, and anti-oxidant enzymes. In contrast, it significantly reduces cell viability, tumor volume, oxidative markers, and NF-κB compared to single therapies (48).

***Thymoquinone and docetaxel***

Therapeutic resistance of cancer cells prevents docetaxel (DTX), the primary drug used to treat prostate cancer. Because DTX with natural ingredients can reduce the required dosage, various studies have combined DTX with herbal ingredients, including thymoquinone. Multiple studies have concluded that combined DTX and thymoquinone minimize side effects and doses of DTX and induce apoptosis more effectively.

One study showed that using thymoquinone and DTX in prostate cancer cells (DU145 and C4-2B) by inhibiting PI3K/AKT signaling reduced cancer cell survival and proliferation with fewer side effects. This study showed that combining DTX with thymoquinone in lower concentrations could induce apoptosis and have a more significant cytotoxic effect on prostate cancer cells, reducing the required dosage (49). Another study on prostate cancer cell lines demonstrated that this combined effect in a dose-dependent manner leads to significant cellular cytotoxicity and induction of more apoptosis compared to any single agent alone (50). 

The combination of DTX and thymoquinone in the mouse model and cell lines of breast cancer that were encapsulated in different nanoparticles showed that this combination could stimulate apoptosis more effectively by inducing DNA damage, leading to increased cytotoxicity, reduced side effects, and reduced chemical drug doses (50, 51). 

***Resveratrol***

Resveratrol is a natural polyphenol and an active ingredient in blueberries that acts as an anti-oxidant, anti-inflammation, anti-oxidation, and anti-cancer. Resveratrol’s antitumor effects include reducing cell proliferation with decreased CXCR4, cyclin D1, cyclin E1, and decreased dihydrotestosterone in prostate cancer cells (LNCaP) (Figure 5). Moreover, some studies showed that combining resveratrol with other anticancer drugs could increase their effects with lower side effects (20). Resveratrol can reduce cell survival by decreasing D1/D2 cyclin and Wnt gene expression in colorectal carcinoma (Figure 5) (52). Also, resveratrol can induce apoptosis by activating the p38 MAPK pathway and suppressing FOXO3a in Benign prostatic hyperplasia epithelial cell line (BPH-1) and metastasis inhibition with suppression of MMP-3 and MMP-9, inhibition of VEGF, and decreased E-cadherin in colorectal cancer cells (53).

***Resveratrol and Raloxifene***

It was shown that the synergistic effects of resveratrol and Raloxifene could increase apoptosis. In a study on MCF7 and MDA-MB-231 breast cancer cells, the combined use of resveratrol and Raloxifene increased Bax, p53, caspase 3, and caspase 8. It decreased Bcl-2 expression, significantly causing apoptosis and reducing the viability of these cells (Figures 3 and 4) (54).

***Resveratrol and 5-FU***

5-Fluorouracil is a common chemical drug used to treat various cancers; however, its main limitations are increased drug resistance and severe toxicity in clinical conditions. The combination of 5-FU and resveratrol in multiple studies showed that this combination could significantly increase the percentage of apoptotic cells by increasing the Bax/Bcl-2 ratio (Figure 3). 

A study showed that combining resveratrol and 5-FU in colorectal cancer cells (DLD1 and HCT116) induced anticancer activity by simultaneously inhibiting the STAT3 and Akt signaling pathways. This study showed that resveratrol could increase the anti-telomeric and pro-apoptotic potentials of 5-FU in colorectal cancer, thus leading to hypersensitivity to chemotherapy (55). 

Resveratrol and the 5-FU combination could cause an arrested cell cycle in the S phase, increase caspase 3 level, induce apoptosis, and inhibit cell growth in a cell line and a mouse skin cancer model (Figure 4-5) (56). Also, using this compound in the breast cancer model has inhibited the YB-1/EZH2 signaling axis and reduced the invasion of cancer cells (57). Other studies showed that the synergistic effects of resveratrol and 5-FU increased apoptotic events and decreased the dose of the chemical drug in the cell line and mouse models of colorectal cancer (58). This combination in colorectal cancers also showed that resveratrol could potentiate the antitumor effects of 5-FU by inhibiting the epithelial-mesenchymal transition (EMT) factor by regulating intercellular junctions and the NF-κB pathway (59).

 In another study, the effect of resveratrol on radiation sensitivity and the use of 5-FU in MCF-7 cell culture was investigated. This study showed that combination therapy with 5-FU, resveratrol, and radiation significantly reduced the spheroid formation ability compared to single treatments (60). In addition, resveratrol significantly reduces drug resistance by inhibiting epithelial-mesenchymal transition (EMT) factors and reducing NF-κB activation. A combination of resveratrol and 5-FU with PEGylated liposomes enhanced the cytotoxicity of the NT8e head and neck cancer cell line (61). This compound also has anti-angiogenic properties. A model of B16 melanoma in mice showed that treatment with resveratrol and 5-FU reduced tumor growth compared to the single-use. This growth inhibitory effect was associated with changes in AMPK, VASP, and VEGF expression (62).

***Resveratrol and doxorubicin***

Doxorubicin and resveratrol have been used to reduce doxorubicin resistance and side effects. *In vivo* studies in a xenograft mouse model of breast cancer have shown that the combination of resveratrol and doxorubicin reduces cardiac toxicity of doxorubicin and its side effects by increasing superoxide dismutase (SOD) and decreasing ROS. Therefore, it increases antitumor activity and has sound protective effects (63). In one study, the ability of resveratrol in combination with doxorubicin to inhibit angiogenesis *in vitro* and *in vivo* was investigated. The combination of resveratrol and doxorubicin significantly inhibited dose-dependent angiogenesis, and their results showed that this combination is a new strategy for preventing and treating angiogenesis (64). 

***Resveratrol and paclitaxel***

It has been suggested that since resveratrol and paclitaxel have different effects on apoptosis and cell cycle regulation, their combination may have synergistic anticancer activity. The study of the combined effect of the two on lung cancer cells (A549, EBC-1, Lu65), glioblastoma cancer cells (DBTRG), and liver cancer cells (HepG2) was performed. The results show that resveratrol significantly increases paclitaxel’s anti-proliferative and apoptotic effect on lung cancer cells (65).

DBTRG glioblastoma cells increase excessive Ca2 + uptake through TRPM2 channel activation, leading to mitochondrial dysfunction and intracellular ROS production, ultimately promoting cancer cell death and Reducing cell survival *in vitro* (66). 

In liver cancer cells (HepG2), resveratrol and paclitaxel combination significantly increased the expression of caspases 3,8,9, BAX, P53, P21, TIMP-1, and TIMP-2 and decreased Bcl-2, NF-KB, and VEGF compared to the control groups (Figures 3 and 4) (67). In addition, resveratrol enhances the response to treatment in paclitaxel-resistant breast cancer cells (MDA-MB-231), reducing cell proliferation and colony formation and increasing apoptosis in these cells (68). Therefore, it can be concluded that resveratrol can be used as an adjunct to paclitaxel to enhance its anticancer effects, and this combination may be used in future clinical applications. 

***Resveratrol and cisplatin***

Numerous studies have shown the effects of the combined use of cisplatin and resveratrol in different cancer models. This compound induces increased apoptosis through other mechanisms and inhibits tumor cell growth by activating the PERK /eIF2α signaling pathway. In a study, the combined use of cisplatin and resveratrol showed that cisplatin toxicity in human hepatocellular carcinoma cells (SMCC7721) increased through an apoptosis-dependent mechanism and increased cancer cell destruction. This combination has also led to ROS production and increased DNA damage. 

In addition, cisplatin and resveratrol synergistically inhibited glutamine metabolism by reducing ASCT2 (a glutamine transporter) and forming γH2AX foci (69). Another study showed that combination therapy with cisplatin and resveratrol increased Bax/Bcl-2 ratio, ROS production, and mitochondrial membrane depolarization and ultimately synergistically increased apoptosis in malignant mesothelioma cells (Figure 3) (70). The efficacy of resveratrol and cisplatin combination therapy in prostate cancer cells was also analyzed. The results showed that resveratrol cooperates with cisplatin in regulating DUSP1 levels and promoting apoptosis, indicating that DUSP1 is a significant determinant of cisplatin sensitivity to apoptosis (71). Another study showed that in ovarian cancer cell line and cisplatin A2780CisR-resistant subclone, resveratrol in combination with cisplatin could improve the effect of cisplatin on ovarian cancer (72). 

***Oxymatrine***

Oxymatrine is a quinolizidine alkaloid compound extracted from the root of *Sophora flavescent*; its anticancer activities were approved (Figure 2). Oxymatrine effects on lung cancer cells (A549) showed inhibition of the binding of STAT5 signaling protein to DNA, and reduction of Bcl-2, Bcl-xl, VEGF, MMP-9, and viability of cancer cells (Figure 3). Their study suggests combining oxymatrine with Paclitaxel inhibits lung cancer cell growth *in vitro* and *in vivo*. Therefore, oxymatrine could be a good candidate for a novel therapeutic agent that effectively treats various malignancies (10). Oxymatrine also has a potent antitumor effect against U251MG glioblastoma cells. Oxymatrine effectively inhibits the proliferation and invasion of Glioblastoma cancer cells by inducing cell cycle arrest in G1 and S phases, reducing D1 and CDK4/6 cyclins, and reducing EGFR expression (Figure 5). It was found that the combination of oxymatrine and Erlotinib (a chemotherapeutic agent for tumors) had a more potent effect on the survival of glioma cells (88). The impact of oxymatrine on gastric cancer cell lines inhibits the IL-21R-mediated JAK2/STAT3 pathway, preventing tumor cell proliferation and invasion (89). Researchers have shown that oxymatrine reduces the viability of T24 bladder cancer cells by increasing Bax and Caspase 3 expression and decreasing the expression of Bcl-2, survivin, and p53 (Figures 3 and 4) (89). 

***Oxymatrine and doxorubicin***

Doxorubicin is a powerful chemotherapeutic agent that effectively treats various types of tumors. However, one of the significant concerns and limitations in its clinical application is the dose-dependent cardiac toxicity in patients. Doxorubicin treatment significantly increases oxidative stress, and the resulting apoptosis damages heart tissue.

Zhang *et al*. investigated the effects of oxymatrine on doxorubicin-induced cardiac toxicity in rat hearts and H9c2 cells. Their results showed that pretreatment with oxymatrine reduced doxorubicin-induced heart damage by reducing oxidative stress, cardiac apoptosis, and LDH and CK-MB expression levels in treated mice. According to this study, oxymatrine may be a promising treatment candidate to help prevent doxorubicin-induced cardiac toxicity (90).

***Oxymatrine and cisplatin***

Combining oxymatrine with cisplatin is one of the strategies used in treatment to overcome drug resistance and reduce the dose of chemical drugs. A study of small cell lung cancer (NSCLC) and Lewis lung cancer xenograft tumor (LLC) showed that oxymatrine and cisplatin increased CD8 T cells and cytokines, including IFN-γ, TNF-α, and IL -2. It also regulates miR-155 and SOCS1. As a result, the combination of oxymatrine and cisplatin can inhibit cell growth and significantly increase antitumor cellular immunity (73). This combination therapy also reduces chemical resistance in BGC823 gastric cancer cells by arresting the cell cycle in the G0/G1 phase by increasing p21 and p27 and decreasing the expression of cyclin D1, producing ROS inactivating the AKT / ERK signaling pathway (Figures 3 and 5). The results also showed that this combination significantly reduced Ki67, p-AKT, and p-ERK in mice with xenografts BGC823 tumor compared to oxymatrine or cisplatin alone. This study showed that concomitant treatment with oxymatrine and cisplatin exerts synergistic antitumor effects on gastric cancer cells (83). 

***Oxymatrine and bevacizumab***

Bevacizumab is a monoclonal antibody against vascular endothelial growth factor A (VEGF-A) and has a potent anti-angiogenic activity used in many cancers. But activating the Wnt / β-Catenin pathway increases cancer stem cells and cancer cells’ invasive and metastatic properties. A study of breast cancer cells (TNBCs) and xenograft mice showed that oxymatrine increased the antitumor effects of Bevacizumab and reduced the risk of recurrence and metastasis by decreasing the ability of TNBC stem cells to regenerate (91).

***Oxymatrine and oxaliplatin***

Oxaliplatin and oxymatrine combination affects the cell cycle, p-PI3K, and p-AKT pathways with better antitumor effects. A study of colorectal cancer *in vitro* and *in vivo* showed that oxymatrine synergistically increased the antitumor activity of oxaliplatin. This combination, on colon cancer cells HT29 and SW480, leads to cell cycle arrest in the G0/G1 phase, increase in p21 and p27, decrease in cyclin D, and induction of apoptosis through a reduction in p-PI3K, p-AKT, and p-mTOR expression, which result in significant tumor volume reduction (Figures 3 and 5) (92). 

***Eugenol***

Eugenol is an active ingredient in cinnamon and a member of the allylbenzene class. It has anti-oxidant, anti-inflammatory, and anticancer properties. The toxic long-term side effects of the chemotherapeutic agent result in using the herbal compound to alleviate these effects. Eugenol was used as an anti-metastatic and anti-proliferative agent against MDA-MB-231 and SK-BR-3 breast cancer cells. Eugenol has shown an anti-breast cancer effect by targeting the caspase pathway and increased apoptosis via enhancing the expression of Caspase3, Caspase7, and Caspase9 (Figure 4). In breast cancer MDA231 cells, Eugenol reduced tumor invasion by reducing MMP2 and MMP9 and increasing collagen-IV and TIMP-1 (93). Eugenol has anti-proliferative and pro-apoptotic effects on various breast cancer cells *in vivo* and *in vitro*. It decreased the viability of breast cancer cells (MCF7) by reducing the expression of E2F1/survivin, inhibiting cyclin D1 and NF- κB, and increasing Bax and Caspase 3 (Figures 3–5) (86). 

***Eugenol and Dacarbazine***

The combination of Eugenol and Dacarbazine increases apoptosis and inhibits cancer cell metastasis. A study on the SK-MEL-28 melanoma cell line found that this combination reduced the proliferation and migration of cancer cells and dacarbazine doses. Thus, the combination of Eugenol and Dacarbazine inhibits resistance and invasive metastasis of melanoma more than either treatment alone (81). 

***Eugenol and Gemcitabine***

A study on Hela cells evaluated the effect of Eugenol alone and combined with Gemcitabine. Eugenol showed selective dose-dependent cytotoxicity to Hela cells compared to normal cells. Also, by significantly reducing Bcl-2, COX-2, and IL-1β, this combination showed growth inhibition and increased apoptosis of cancer cells compared to single-use, thus reducing the dose of the chemical drug (Figure 3) (94). 

***Eugenol and cisplatin***

Eugenol increases cisplatin’s anticancer and apoptotic activity against many cancer cells, including HeLa and breast cancer cells (MDA-MB-231, MDA-MB-468, and BT-20) and Human melanoma cells (G361). A study on these cell lines shows that combination therapy with cisplatin and Eugenol increases apoptotic proteins such as caspases and decreases the expression of anti-apoptotic proteins such as Bcl-2 compared to single-use (Figure 3) (95). Combination therapy of cisplatin and Eugenol on a mouse model of breast cancer significantly inhibited NF-κB and reduced Ki-67, N-cadherin, Snail-1, and Sox-2 (96). 

cisplatin/Eugenol treatment in SKOV3 and OV2774 ovarian cancer cell and tumor-bearing mice reduced drug resistance and tumor volume by inhibiting the Notch-Hes1 pathway (74). One of the side effects of chemotherapy drugs such as cisplatin is increased oxidative stress in various tissues, leading to apoptosis and damage to these organs. In two separate studies, Eugenol was investigated for its anti-apoptotic and anti-oxidant effects against cisplatin-induced damage to the testis and middle ear. The findings show that Eugenol reduces the severity of cisplatin-induced injury in targeted tissues by increasing levels of anti-oxidant enzymes and decreasing oxidant parameters (97).

***Rosmarinic acid***

Rosmarinic acid is an active ingredient in rosemary. Rosmarinic has an antitumor role through apoptosis-inducing in human gastric cancer SGC-7901 cells via enhancing Bax and Caspase 3 agents and reducing the expression of Bcl-2 (Figures 3 and 4) (98). Also, rosmarinic acid has antiproliferative effects (99). A study showed that adding rosmarinic acid to DU145 prostate cancer cells reduces the proliferation of tumor cells by inhibiting HDAC2, increasing p53 expression, and reducing the expression of D1 and E1 cyclin (Figures 3 and 5) (100). Rosmarinic acid significantly inhibits metalloproteinase 9 (MMP9) and increases the expression of Collagen-I. It also increases tissue inhibitor metalloproteinases-1 (TIMP-1) on mRNA levels in gastric adenocarcinoma CRL-1739, reducing invasion and migration (57).

***Rosmarinic acid and cisplatin***

The synergistic effect of cisplatin and rosmarinic acid has been investigated in various studies. A study found that this combination increases apoptosis and the susceptibility of cisplatin-resistant cells to apoptosis by arresting the cell cycle in the G2/M phase (Figure 5). The mTOR / S6K1 signaling pathway is critical in cervical cancer, and blocking this pathway leads to degeneration of cervical cancer cells.

Rosmarinic acid (as methyl ester) combined with cisplatin on cervical cancer cells; HeLa, SiHa, and A549, decreased mTOR-mediated activation of S6K1, increased LC3 (autophagy-associated protein), and induced Bax and p53 (Figure 5). Thus, rosmarinic acid sensitizes chemotherapy-resistant cells and increases the antitumor effect of cisplatin (101). A study investigated the effect of rosmarinic acid on drug resistance reduction in small-cell lung cancer (NSCLC). The data showed that rosmarinic acid, in a dose-dependent manner, inhibited NSCLC cell proliferation and colony formation, arrested the cell cycle in the G1 phase, and increased the sensitivity of cisplatin-resistant cells (Figure 5). Also, the growth of NSCLC was significantly inhibited in a xenograft mouse model (102). Another study examined the effect of this combination on renal cell carcinoma and concluded that it interferes with the activity of cancer cells. Also, arresting in the G2/M phase induces apoptosis and cytotoxicity and significantly inhibits the expression of p-FAK (Tyr 925) in RCC 786-O cells (Figure 5) (82).

***Rosmarinic acid and 5-FU***

The combination of rosmarinic acid and 5-FU against SGC7901 gastric cancer cells reduces the expression of miR-6785-5p and miR-642a-3p and increases the expression of FOXO4 and Bax (Figure 3). The data showed that rosmarinic acid increased the sensitivity of SGC7901 gastric cancer cells to 5-FU, and apoptosis was significantly increased in the combined group compared to the single-therapy group (103). 

***Curcumin***

Curcumin is a diarylheptanoid and is an active ingredient of turmeric with a bright yellow color. Several studies have shown that curcumin has anti-oxidant, anti-inflammatory, antiviral, antibacterial, antifungal, and anticancer activities and has the potential against various malignant diseases, diabetes, allergies, arthritis, Alzheimer’s disease, and other chronic illnesses (75).

Curcumin manages oxidative and inflammatory conditions, metabolic syndrome, arthritis, and cancer. One research has shown that curcumin could inhibit the proliferation and induce apoptosis of SGC-7901 gastric cancer cells via increasing the content of miR-34a microRNA, expression of caspases 3, caspase 9, Bid, and Bax, and reducing the expression of Bcl-2 (Figures 3 and 4) (104). It has potential anticancer activity on oral squamous cell carcinoma (OSCC) through inhibition of nuclear factor kappa B (NF κB) and cyclooxygenase 2 (COX-2) expression which is involved in the development of cancer (105). Curcumin exerts its anti-invasive effects by increasing TIMP-1, decreasing MMP-2 and VEGF, and inhibiting cell proliferation in MCF-7 and MDA-MB-231 breast cancer cells (106).

***Curcuminoid and cisplatin***

Curcuminoid WZ35 is a new curcumin analog with anticancer effects *in vitro* and *in vivo*. Combined with cisplatin on SGC-7901 and BGC-823 gastric cancer cells, it significantly increases apoptosis by inhibiting TrxR1 activity, inducing ROS, and activating the p38 and JNK signaling pathways. *In vivo* study showed that tumor growth is significantly suppressed in the gastric cancer xenograft mouse model. This study suggested that combination therapy with curcuminoid WZ35 and cisplatin may be a more effective treatment for gastric cancer (75)E.

Another study investigated WZ26 curcumin analog with cisplatin on a cell line and a mouse model of colon cancer. WZ26, in combination with cisplatin, significantly increases ROS, resulting in DNA damage and activation of the JNK signaling pathway. Also, WZ26 significantly inhibited tumor growth in the xenograft mouse model (107). 

***Diphenyl difluoromethane (EF24) and Mitotane***

EF24 is a curcumin derivative that exhibits more potent biological activity than curcumin. A study showed that EF24 combined with Mitotane on adrenal cortical tumor cell lines (SW13 and H295R) reduced cell viability and migration and arrested the cell cycle in the G1 phase. In addition, EF24b regulates the Wnt/β-catenin and PI3k/Akt pathways and increases the intracellular ROS (87).

***Curcumin and*** ***doxorubicin***

Doxorubicin is an anti-breast cancer agent that has shown resistance during long-term treatments. The study results showed that curcumin could significantly increase the effect of doxorubicin in doxorubicin-resistant cancer cells by inhibiting ATPase activity in MCF7 and MDA-MB-231 human breast cancer cells (79).

In several studies, the combined use of curcumin and doxorubicin has been performed in invasive B cell lymphoma cell lines and a mouse model of neuroblastoma, melanoma, and a mouse model of lung cancer. It was shown that the anticancer activity of this combination was significantly higher than the treatments of curcumin and doxorubicin alone. According to the results, the rearrangement of p53 and p21 and the increase of apoptotic proteins increase the apoptosis rate and have more anticancer effects. This combination inhibits tumor migration and reduces tumor volume (Figure 3) (85, 108).

***Curcuminoid and*** ***oxaliplatin***

The effects of curcumin and its synthetic analogs in combination with oxaliplatin in various malignant cell lines have been studied *in vitro* and *in vivo*. The combination of curcumin and oxaliplatin significantly suppresses colorectal carcinoma by inducing apoptosis, inhibiting cancer growth in mouse models. These effects will be more effective in colorectal cancer if liposomal capsules are used *in vitro* and *in vivo* (109). Evidence showed that the development of many malignancies, such as colon cancer, is associated with activating several signaling pathways, including the EGFR (EGF-receptor) and IGF/IGFR1 pathways, which increase proliferation, inhibit apoptosis, and induce metastasis. 

The results of *in vitro* study on colorectal cancer cells (HCT-116 and HT-29) showed that the effects of combination therapy with curcumin and oxaliplatin are due to attenuation of EGFR (EGF-receptor) and IGF-1R signaling pathways (110). The use of dendrosomal nano-curcumin in combination with oxaliplatin is effective in cell death and induction of apoptosis in human ovarian carcinoma cell lines. Compared to a single treatment field, this combination significantly affects the relative expression of long non-coding RNAs. This combination leads to a significant increase in the induction of apoptosis and inhibition of the growth of xenograft mouse model gastric cancer compared to the control group through caspase 3, 8, 9, and Bax/ Bcl-2 (Figures 3 and 4) (111). Oxaliplatin resistance affects the outcomes of patients with metastatic colorectal cancer treated with this drug. Curcumin overcomes oxaliplatin-resistance in colon cancer through various mechanisms such as regulation of TGF-β, Smad2/3, CXC Chemokine, and NF-κB signaling pathways ERCC1 expression by miR-409-3p and reduction of cancer stem cells (CSC) (112, 113). One main limiting factor in using oxaliplatin in cancer patients is tissue oxidative stress damage induced by oxaliplatin. Studies show that curcumin reduces oxaliplatin-induced liver damage and oxidative stress by activating the Nrf2 pathway. In addition, it relieves peripheral neuropathic pain caused by oxaliplatin by inhibiting NF-κB activation induced by oxidative stress and reducing inflammation (114).

***Gingerol***

Gingerol is an active ingredient in ginger, used to induce hypothermia. It has anti-inflammatory, anti-oxidant, and antiseptic effects and is toxic to many cancer cells (115-117). Gingerol reduces the invasion of MDA-MB-23, as a human breast cancer cell line, by reducing MMP-2 and MMP-9 (118). It decreases cyclin B1 and E1, arrests the cell cycle in the G1 phase, and reduces the proliferation of cervical cancer Hela cells (Figure 5). Additionally, it could inhibit PI3K/AKT and activate AMPK, inducing mTOR-mediated cell apoptosis in HeLa cells (119). Adding Gingerol to RB355 retinoblastoma cell culture in a dose-dependent and time-dependent manner inhibits the PI3K/Akt signaling pathway, arrests the cell cycle in the G2/M phase, and increases caspases 3 and 9, reducing the viability of these tumor cells (Figures 4 and 5) (76). 

***Gingerol and cisplatin***

The combination of Gingerol and cisplatin increases the sensitivity of gastric cancer cells to cisplatin compared to single therapies. A study on HGC-27 and MGC-803 gastric cancer cells showed that this combination arrests the cell cycle in the G1 phase by decreasing cyclin D1 and A2 and increasing the expression of P21 and P27 (Figures 3 and 5). It also reduces cancer cell migration and invasion by reducing MMP-9 and inhibiting the p-PI3K/AKT signaling pathway. These mechanisms sensitize cancer cells to chemotherapy (120). Another study examined the antitumor activity of this combination on oral cells (SCC4, KB) and cervical cancer (HeLa). The results showed that Gingerol induced dose-dependent cytotoxicity in all three cell lines. Arresting cell cycle in the G2 phase in KB and HeLa cells and S phase in SCC4 cells had better therapeutic effects in oral carcinoma and cervical cancer (Figure 5) (80).

***Gingerol and doxorubicin***

Ginger improves the cytotoxic effects of doxorubicin and reduces its side effects. Compared with a single treatment, the combination of Gingerol and doxorubicin in liver cancer cells (HepG2 and Huh7) significantly arrested the cell cycle in the G2/M phase and reduced doxorubicin-induced vascular toxicity through its anti-oxidant properties (Figure 5) (121). In addition, this combination significantly increases the number of apoptotic cells in breast cancer cells by increasing caspase-3 levels and decreasing Cdk-6 cyclin levels (Figure 4-5).

It also significantly reduces the volume of primary tumors and the number of circulating tumor cells (CTCs) in the xenograft mouse model of breast cancer (122). Studies show Gingerol protects the heart against doxorubicin-induced cardiac toxicity through its anti-oxidant effect, NF-κB modulation, and apoptosis (123). Developing anticancer drug delivery systems has recently been considered to increase stability, targeted delivery, and reduce toxic effects. In one study, the antiproliferative effects of magnetic hydroxyapatite alginate (m-HAP) nanocarrier containing Gingerol and doxorubicin on MCF-7 and HEpG2 cells were investigated, and the results of this study showed that this system has a profound anti-proliferative effect in killing cancer cells compared to control groups (124).

***Thujone***

Thujone is an active ingredient in common sage and is a natural ketone. It acts as an antagonist of γ-aminobutyric acid type A (GABA). CD3AK cells effector CD8 T cells in tumor immunotherapy have shown their proliferation could be promoted by α-Thujone (125). This study revealed that even with a low concentration, α-Thujone could increase NK cells’ cytotoxicity by increasing the expression of CD107a, p-Akt, and p-ERK1/2 in colon cancer cells (HCT116) (126). In the presence of Thujone, the A375 melanoma cell culture loses its viability due to reduced expression of Bcl-2, increased expression of Bax, caspase-3, and cytochrome field release (Figures 3 and 4) (15). The *in vitro* and *in vivo* studies have shown that Thujone reduced the main angiogenic factors, including VEGF and angiopoietin 4, and induced apoptosis in glioblastoma cells (15). No analysis was found on the combination of Thujone and chemotherapeutic drugs.

**Table 1:** Anticancer effects of active ingredients of herbal extracts. Different antitumor mechanisms including apoptosis, cell cycle arrest, anti-angiogenesis, anti-metastatic, antioxidant, anti-proliferation were mentioned for each active ingredients

Active Ingredient	Antitumor mechanism	Reference
Noscapine	apoptosis induction, cell cycle arrest, anti-angiogenesis, anti-metastatic	(7)
Thymoquinone	decreased angiogenesis, antioxidant, cell cycle arrest, apoptosis induction	(8)
Resveratrol	apoptosis induction, anti-metastatic, anti-proliferation	(9)
Oxymatrine	apoptosis induction, anti-metastatic, cell cycle arrest	(10)
Eugenol	anti-metastatic, apoptosis induction	(11)
Rosmarinic acid	apoptosis induction, anti-metastatic, anti-proliferation, cell cycle arrest	(11)
Curcumin	apoptosis induction, anti-metastatic, anti-proliferation	(12)
Gingerol	anti-metastatic, cell cycle arrest, signaling pathway editing	(13, 14)
Thujone	apoptosis, anti-angiogenesis, nk cell cytotoxicity induction	(15, 16)

**Table 2:** Drugs used in combination therapy. Chemotherapy drugs with different mechanisms in combination with various active ingredients of herbal medicine were listed

Drug	Mechanism of action	Combination with
cisplatin	DNA alkylation	Noscapine (24, 28), Thymoquinone (39, 41), resveratrol (70), Oxymatrine (73), Eugenol (74), Rosmarinic acid (57), Curcumin (75), Gingerol (76)
Docetaxel	Microtubule inhibitor	Noscapine (77), Thymoquinone (50)
Paclitaxel	Mitosis inhibitor	Noscapine (26), resveratrol (65)
Doxorubicin	Topoisomerase II inhibitor	Noscapine (28), Thymoquinone (43, 45), resveratrol (63), Oxymatrine (78), Curcumin (79), Gingerol (80)
Gemcitabine	pyrimidine analogs	Eugenol (81)
Fluorouracil (5-FU)	Thymidylate synthase inhibitor	Thymoquinone (34), resveratrol (59), Rosmarinic acid (82)
Cyclophosphamide	DNA alkylation	Thymoquinone (37)
Raloxifene	Estrogen antagonists	Resveratrol (53)
Rapamycin	Inhibits Lymphocytes activation	Resveratrol (62)
Bevacizumab	Angiogenesis blocker by VEGF inhibition	Oxymatrine (83)
Oxaliplatin	Inhibition of DNA synthesis	Oxymatrine (84), Curcumin (85)
Dacarbazine	DNA alkylation	Eugenol (86)
Mitotane	Adrenal cortex inhibitor	Curcumin (87)

Nos: noscapine; Thymo: Thymoquinone; Res: resveratrol; Oxy: Oxymatrine; Eug: Eugenol; Ros: Rosmarinic acid; Cur: Curcumin; Gin: Gingerol; Thu: Thujone; Cis: cisplatin; Pac: Paclitaxel; Dox: Doxorubicin; 5-FU: 5- fluorouracil; Oxal: Oxaliplatin; Ralox: Raloxifene; Gem: Gemcitabine

Nos: noscapine; Thymo: Thymoquinone; Res: resveratrol; Oxy: Oxymatrine; Eug: Eugenol; Ros: Rosmarinic acid; Cur: Curcumin; Gin: Gingerol; Thu: Thujone; Cis: cisplatin; Pac: Paclitaxel; Dox: Doxorubicin; 5-FU: 5- fluorouracil; Oxal: Oxaliplatin; Ralox: Raloxifene; Gem: Gemcitabine

Nos: noscapine; Thymo: Thymoquinone; Res: resveratrol; Oxy: Oxymatrine; Eug: Eugenol; Ros: Rosmarinic acid; Cur: Curcumin; Gin: Gingerol; Thu: Thujone; Cis: cisplatin; Pac: Paclitaxel; Dox: Doxorubicin; 5-FU: 5- fluorouracil; Oxal: Oxaliplatin; Ralox: Raloxifene; Gem: Gemcitabine

## Conclusion

Selected studies showed the anticancer effects of active herbal ingredients by inducing apoptosis and cell cycle arrest in cancer cells. These studies also showed that herbal compounds could reduce side effects and dosage, potentiate anticancer responses, and sensitize the cancer cell to chemotherapy drugs. Also, based on the anti-tumor results obtained from noscapine and thymoquinone, these are the most frequent and widely used cases in anti-cancer treatment and can be used together with chemotherapy drugs. 

## Authors’ Contributions

R HB^,^ P N, and N K conceived the study, performed data processing and collection, experiment analysis, and interpretation of results. AM S helped with draft manuscript preparation and visualization. N HR and K G critically revised or edited the article. G M and A G approved the final version to be published. A G supervised and helped acquire funds.

## Limitations

The only limitation of the selected studies was insufficient clinical trials in this subject.

## Perspective and Future Direction

To design a new therapeutic approach in the future, the combination of other herbal active ingredients and chemotherapeutic agents could be assayed to reveal their basic mechanisms. Also, based on the promising benefits of the studied components, their clinical trials in combination with chemotherapy agents are suggested. 

## Conflicts of Interest

The authors report no conflicts of interest.
